# Elevation‐dependent tree growth response to climate in a natural Scots pine/downy birch forest in northern Sweden

**DOI:** 10.1002/pei3.10140

**Published:** 2024-04-01

**Authors:** Magdalena Fassl, Tuomas Aakala, Lars Östlund

**Affiliations:** ^1^ Department of Forest Ecology and Management Swedish University of Agricultural Sciences Umeå Sweden; ^2^ School of Forest Sciences University of Eastern Finland Joensuu Finland

**Keywords:** *Betula pubescens*, climate–growth relationships, dendroecology, Fennoscandia, old growth, *Pinus sylvestris*, Tjeggelvas nature reserve, tree rings

## Abstract

Forests dominate the landscape at high latitudes in the boreal regions and contribute significantly to the global carbon stock. Large areas are protected and provide possibilities to analyze natural forest dynamics including resilience to climate change. In Fennoscandia, Scots pine (*Pinus sylvestris* L.) and downy birch (*Betula pubescens* Ehrh.) often coexist in natural forests close to the limits of their ecological ranges. Tree growth in these forests is generally thought to be limited by temperature, and changes in growth trends can therefore serve as early indicators of the impact of global warming on natural ecosystems. We sampled 592 Scots pine and downy birch trees along two elevational gradients spanning the transition from the forest zone to the coniferous treeline in Tjeggelvas nature reserve, northern Sweden. Based on the tree‐ring data, we compared annual basal area increment (BAI) trends from 1902 to 2021, analyzed the ring‐width indices (RWI) in relation to local climate data, and investigated trends in climate–growth relationships. We found that the mean annual growth of both species was higher in more recent years than at the beginning of the 20th century. The RWI were positively correlated with summer temperatures, however, we found a much stronger relationship for Scots pine than downy birch. We noticed a decrease in the importance of summer temperature for Scots pine growth, whereas the importance of late spring temperatures increased over the 120‐year‐long study period. Due to strongly positive BAI trends combined with a decrease in temperature sensitivity, the overall conclusion of our study is that the influence of increasing temperatures is still positive and outweighs the negative impacts of climate change on Scots pine growth in natural forests in northern Sweden, particularly at higher elevations. Natural forests are important natural experiments that contrast the managed forests and are key to understanding the latter.

## Graphical abstract

Changes in tree growth trends in protected natural forests in northern Fennoscandia can serve as early indicators of the impact of global warming on natural ecosystems. We took increment cores from a total of 592 Scots pine and downy birch trees along an elevational gradient, calculated the basal area increments and investigated trends in climate–growth relationships during the 120‐year‐long study period (1902–2021) based on the annual tree‐ring widths. Our results suggest that Scots pine trees growing in mixed natural forests, particularly at higher elevations, still benefit from global warming.

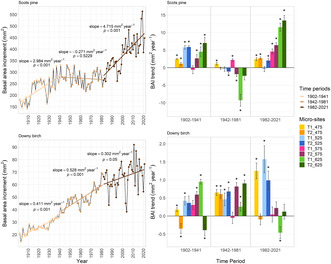

## 1 INTRODUCTION

Boreal forests dominate the landscape at high northern latitudes. They have a greater influence on the mean global temperature than any other biome; changes in land cover in boreal forests strongly affect the surface albedo and therefore the balance of solar radiation (Snyder et al., 2004). They contribute significantly to the global carbon stock, accounting for approximately 32% of the total carbon stored in forests around the world (Pan et al., 2011).

It is challenging to determine the primary factors that influence the structure and growth of boreal forests due to the complex interplay among different variables such as climate, nutrient availability and soil moisture (Bonan & Shugart, 1989). However, the locations of treelines around the globe evidence the temperature dependency of trees growing close to their distribution ranges, as they typically occur at elevations where the average temperature during the growing season is 6.4 ± 0.7°C (Körner, 2012). Global warming is amplified at high elevations and latitudes (Pepin et al., 2015; Rantanen et al., 2022; Serreze & Barry, 2011) and we can, therefore, expect longer growing seasons, a corresponding increase in tree growth in the subarctic as well as a northward advance of the boreal forest into the tundra. This will change the treeline ecotone and hence affect the biodiversity, microtopography, and plant and animal communities (Wielgolaski et al., 2017).

The correlation between temperature and annual tree‐ring width is particularly strong in Scots pine trees in northern Fennoscandia (Grudd et al., 2002) growing close to their thermal limit of distribution. As a result of this dependency of growth on temperature, these trees can serve as early indicators of the effect of global warming on the boreal forest ecosystem (Franke et al., 2017). While tree‐ring patterns of Scots pine (*Pinus sylvestris* L.) in relation to climate have been explored, the focus has been on dominant trees to reduce nonclimatic influences on growth for most dendroclimatological studies (Fritts, 1976). Due to this selective sampling method, the resulting tree‐ring data are not necessarily representative of entire forests, and even less so for mixed forest systems (Nehrbass‐Ahles et al., 2014).

The northernmost forests in Fennoscandia are to a large extent natural forest ecosystems and make up the majority of the 8.8% of formally protected forests in Sweden (SCB, 2022). In contrast to the intensively managed production forests further south, these natural forests are characterized by the presence of ancient trees, structural heterogeneity, and a mix of tree species and are influenced primarily by natural disturbance dynamics such as fire and wind (Kuuluvainen et al., 2017).

Investigating the growth response of both Scots pine and downy birch (*Betula pubescens* Ehrh.) trees to climate change across different sites in a natural forest is especially relevant since recent research based on National Forest Inventory (NFI) data has shown that overall tree growth in Sweden started to decline in 2012 after many decades of positive growth trends (Fridman et al., 2022). While both the cause of this sudden change and the effects of modern management practices on the ecosystem service supply of the boreal forest are unclear (Pohjanmies et al., 2017), it is known that large changes in the northern forest ecosystems are often triggered by the interplay of climatic‐ and human‐induced changes (Chapin et al., 2004). However, the drivers of this growth decline in managed forests might not apply to natural forests with more uneven age structures and little to no human influence. Understanding the growth dynamics of these natural forests is crucial for enhancing the adaptability of managed forests to the challenges imposed by climate change.

A number of studies have addressed the dendroclimatology of northern Fennoscandia. Primarily, the focus has been on temperature reconstruction, with the longest chronologies covering millennia (Helama et al., 2002). However, these studies tend to focus on maximizing the climate signal and/or the temporal coverage, and thus are not necessarily representative for how the trees in the forested areas are responding to climate variability (Aakala et al., 2018). As the region is undergoing rapid changes in climate, differences in climate–growth relationships among species mean shifts in their competitiveness. Thus, understanding how the climate response differs among the main tree species in the forests provides understanding necessary to predict how the ecosystems are likely to develop.

Our study aims to provide insights into changes in Scots pine and downy birch growth in a protected subalpine forest in Tjeggelvas nature reserve (TNR) that experiences very little anthropogenic disturbance. To achieve this, we used tree‐ring data collected along an elevational gradient ranging from the forest zone (475 m.a.s.l.) to the coniferous treeline (625 m.a.s.l.) to calculate annual growth, expressed as basal area increment (BAI), and analyzed the tree‐ring widths in relation to local temperature data from 1902 to 2021. We aimed to answer the following questions:

- How did the BAI of Scots pine and downy birch change over the studied time period?
- How did the importance of temperature and precipitation for the growth of both species vary depending on the elevation?
- How did these climate–growth relationships change over time?

## 2 METHODS

### 2.1 Study area and sample collection

**FIGURE 1:**
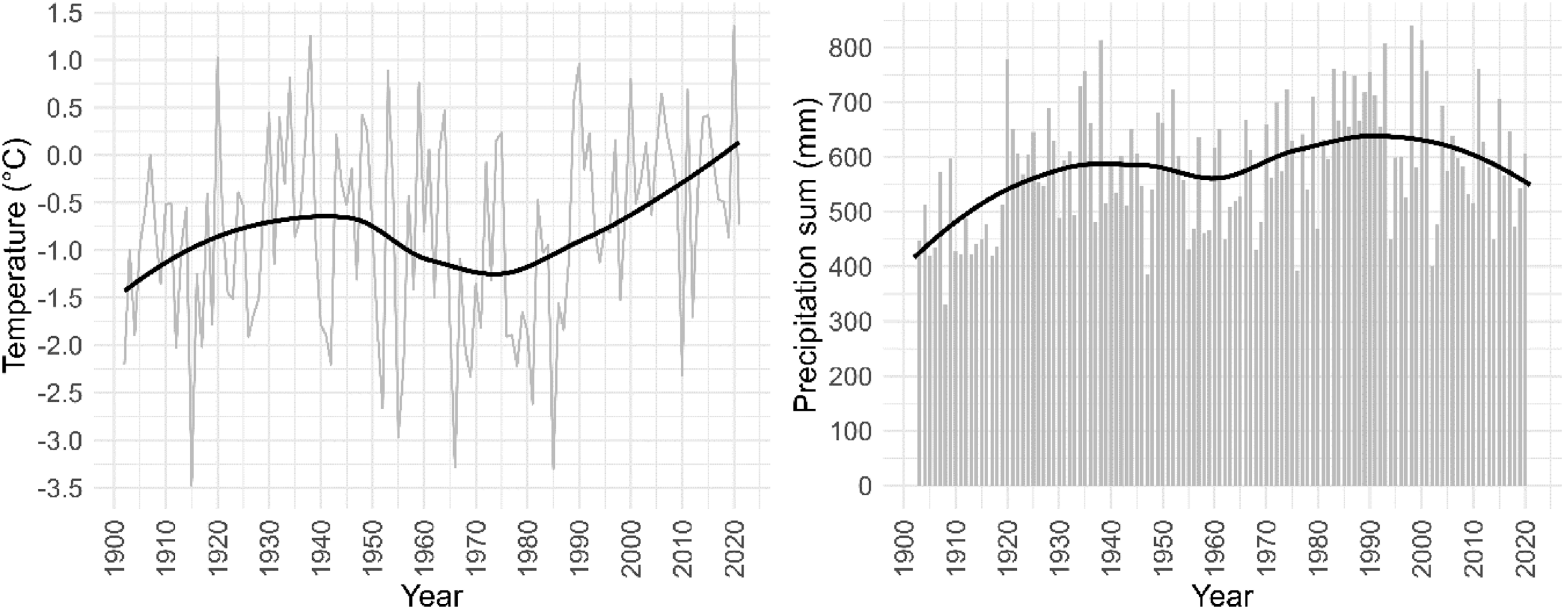
Mean annual temperature and precipitation sum including trend lines (bold) from Kvikkjokk–Årrenjarka for 1902–2021.

Our study area is located in Tjeggelvas nature reserve (66.62° N, 17.57° E; 300 km^2^), approximately 60 km north of Arjeplog in Swedish Sápmi, the traditional territory of the indigenous Sámi people. The climate in this area is classified as subarctic in the Köppen–Geiger system (Beck et al., 2018). The closest climate station is located 35 km from the study area in Kvikkjokk–Årrenjarka (313 m.a.s.l.; data available from 1886 to today), where the annual mean temperature is −0.9°C with a mean precipitation sum of 565 mm. During the summer months (June–September), the mean temperature is 10.2°C with 272 mm of precipitation. Figure 1 shows the mean annual temperature and precipitation sums.

Tjeggelvas nature reserve has never been commercially logged and was largely spared when the timber frontier arrived in the early 20th century (Cosatti, 2022) led to a decrease in the number of large trees in most other parts of northern Sweden through high‐grading (Östlund & Norstedt, 2021). However, the region has been inhabited by the Sámi people since time immemorial and traces of their low‐impact land use for subsistence (e.g., bark‐peelings, reindeer fences, lichen stumps) can be found throughout the forests in TNR (e.g., Norstedt et al., 2017; Östlund & Bergman, 2006; Rautio et al., 2014). Reindeer herding is still practiced today, though less intensively compared to prior to the end of the 19th century (Josefsson et al., 2010).

At lower elevations close to Lake Tjeggelvas (~450 m.a.s.l.), TNR is characterized by late‐successional forests dominated by Scots pine (*P. sylvestris* L.) and downy birch (*B. pubescens* Ehrh.) with a minor component of rowan (*Sorbus aucuparia* L.) and goat willow (*Salix caprea* L.) growing on glacial till. The forest landscape is interspersed with streams, mires and granite boulder fields, and the ground vegetation consists mainly of dwarf shrubs (*Vaccinium* spp., *Empetrum* spp.) and lichens and mosses such as *Cladonia rangiferina* (L.) Weber ex F.H. Wigg (1780) and *Pleurozium schreberi* (Brid.) Mitt. The Scots pine treeline is located at approximately 625 m.a.s.l., the downy birch treeline at 725 m.a.s.l. Beyond the deciduous treeline and up to the bare mountain tops, the landscape is dominated by mountain heath.

**FIGURE 2:**
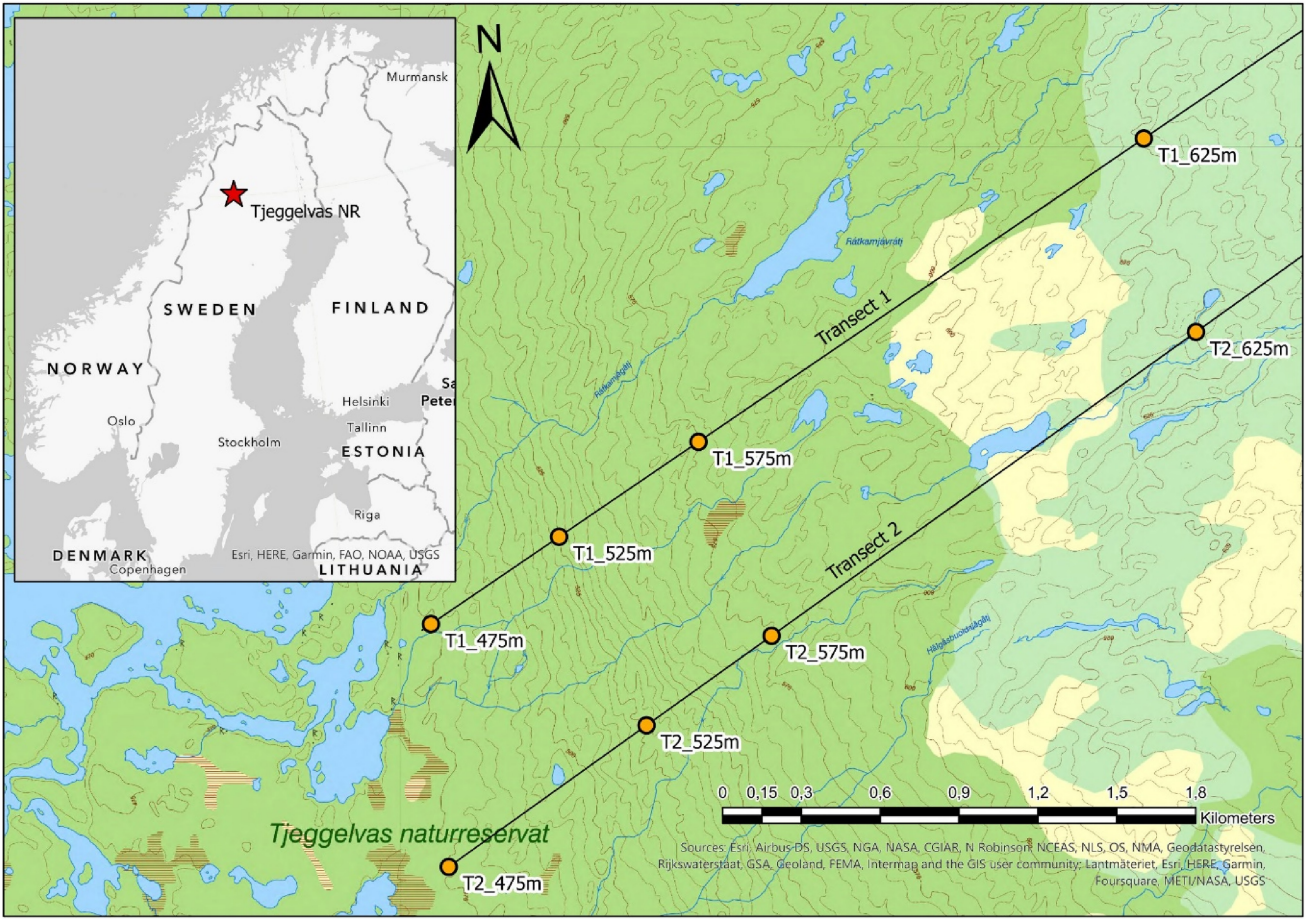
Locations of the micro‐sites in Tjeggelvas nature reserve. Darkgreen areas are dominated by Scots pine, lightgreen areas by downy birch, pale yellow areas are the locations of big boulder fields, and hatched areas are mires. The inset figure shows the location of our study area in Fennoscandia.

For the fieldwork, we randomly selected two transect lines perpendicular to the west‐facing slope (approximately 750 m apart) and established four micro‐sites per transect line across the elevational gradient (Figure 2). We used a density‐based sampling approach (Jonsson et al., 1992) and aimed to core 40–60 Scots pine and approximately 20 downy birch trees per micro‐site. The centers of the circular micro‐sites were located at approximately 475, 525, 575, and 625 m.a.s.l. As their sizes depended on the density of the forest at each location, the radii ranged from 20 to 50 m.

Within each micro‐site, we measured the basal areas with a relascope and took one core at breast height (1.3 m above ground) from each living Scots pine with a diameter at breast height (DBH) ≥10 cm and each living downy birch with a DBH ≥8 cm with a standard 5.15 mm increment borer. To minimize the influence of reaction wood that commonly forms in trees growing on undulating terrain (Schweingruber, 1988) on our analyses, we cored parallel to the contour line, that is, perpendicular to the slope.

We prepared the samples following standard dendrochronological procedures (Fritts, 1976; Schweingruber, 1988): we air‐dried and glued them to wooden supports before sanding them with progressively finer sandpaper (240‐ to 1000‐grit) until the tree‐ring boundaries were clearly visible.

### 2.2 Dendrochronological analyses

After scanning the samples with a high‐resolution (3200 dpi) flatbed scanner, we measured the tree‐ring widths (TRW) with CooRecorder 9.8.1 (Larsson & Larsson, 2022) to a precision of 0.01 mm. Since poorer growing conditions at higher latitudes can increase the frequency of missing rings (Wilmking et al., 2012), we crossdated each TRW measurement series with a local, species‐specific master chronology in CDendro 9.8.1 (Larsson & Larsson, 2022) and assigned the corresponding calendar year to each ring. We had to exclude 41 out of the 592 tree cores that we took from both the basal area increment and the climate–growth correlation analyses because they were too rotten or broken into too many pieces to allow for accurate dating (Table 1).

**TABLE 1:** Description of the micro‐sites.

Micro‐site	Elevation (m.a.s.l.)	Basal area (m^2^ ha^−1^) of living trees (Scots pine/downy birch)	Calculated number of stems per hectare (Scots pine/downy birch)	Number of cores in total (Scots pine/downy birch)	Number of excluded cores (Scots pine/downy birch)	Number of cores included in the BAI analyses (Scots pine/downy birch)	Number of cores included in the climate–growth analyses (Scots pine/downy birch)	Mean age (±SD) at breast height (Scots pine/downy birch)	Starting year of micro‐site chronology (Scots pine/downy birch)
T1_475	475	6/1	229/178	45/25	5/3	37/17	40/4	140 (±40)/102 (±31)	1815/1860
T2_475	475	10/0	216/71	61/10	4/1	50/9	57/2	182 (±88)/103 (±31)	1689/1861
T1_525	525	12/1	205/134	58/28	2/3	45/20	41/4	130 (±42)/105 (±28)	1540/1850
T2_525	525	5/0	239/50	47/22	1/2	32/19	46/3	125 (±102)/84 (±27)	1505/1889
T1_575	575	13/0	219/294	43/27	2/3	28/20	41/4	138 (±136)/114 (±33)	1469/1840
T2_575	575	5/0	107/106	54/42	4/1	42/17	50/5	155 (±82)/105 (±27)	1559/1872
T1_625	625	8/6	80/481	63/24	1/3	58/20	31/4	70 (±51)/102 (±28)	1801/1870
T2_625	625	1/0	39/56	30/34	0/6	30/26	31/2	94 (±50)/95 (±28)	1810/1854

*Note*: The mean age was calculated from complete cores only and therefore reflects the mean age of trees without rotten centers rather than the mean age of all trees in the micro‐sites. Basal areas were measured from the centers of the micro‐sites. A basal area of “0” does not mean that there were no birches in a micro‐site but rather that the trees had small diameters and/or were located closer to the edges of the micro‐sites and were therefore not picked up by the relascope measurements. We calculated the number of stems per hectare based on the number of living trees in each micro‐site. More detailed information about the micro‐site radii, the number of trees per micro‐site, and the dominant ground vegetation can be found in the supplementary material (Table S1).

Abbreviation: BAI, basal area increment.

All data analyses were conducted in the R statistical environment (R Core Team, 2022). We used the packages dplR (Bunn & Korpela, 2020) and treeclim (Zang & Biondi, 2015) for the tree‐ring analyses, as well as dplyr (Wickham et al., 2022) for data manipulation, and ggplot2 (Wickham, 2016) and gridExtra (Auguie & Antonov, 2017) to create the figures.

### 2.3 Basal area increment and climate–growth correlation analyses

We calculated the basal area increment, that is, the ring area, for each tree and year since it is a better measure for growth than the raw tree‐ring width (Biondi & Qeadan, 2008) and also reduces age‐related growth trends (Bista et al., 2021). We used the bai.out() function in dplR which calculates the BAI series following this formula:

$${\mathrm{BAI}}_{t}=\pi \left({w_{t}}^{2}+2w_{t}R_{t-1}\right),$$

where *w*_*t*_ is the ring width of the current year and *R*_*t* − 1_ is the radius of the stem (excluding the bark) prior to the current year's growing season. After turning the ring width series of every tree into a BAI series, we calculated the species‐specific means for each micro‐site as well as for the entire data set. To model the trends in tree growth over time, we used ordinary least square regression equations with BAI as the dependent and time as the independent variable.

For calculating the climate–growth correlations, it is better to use dimensionless ring‐width indices (RWI) because the formula for calculating BAI values includes the tree radius and because ring width typically displays strong serial autocorrelation. This creates interdependence between the yearly BAI values, which violates the assumption of independence and would lead to incorrect *p*‐values.

Additionally, while calculating basal area increments reduces the age effect, they may still contain long‐term growth trends caused by nonclimatic factors (Rubio‐Cuadrado et al., 2020).

To calculate the RWI, we first detrended all raw ring‐width series to remove the age‐related decline in TRW (Fritts, 1976). We used cubic smoothing spline functions with a 50% frequency cutoff at 30 years fitted to each measurement series for detrending as they retain short‐term variation in ring widths better than stiffer splines (Holmes, 1983). We removed the autocorrelation by using Tukey's biweight robust mean which reduces the effect of outliers (Cook et al., 1990) and averaged the RWI series into prewhitened residual subchronologies for each micro‐site. To analyze the relationships between annual RWI and monthly mean temperature and precipitation sums data from Kvikkjokk climate station, we calculated Pearson's correlation coefficients with significance levels of 95% (*p* < .05) using the dcc()‐function of the treeclim package. Since weather conditions outside of the growing season can also influence tree growth (Fritts, 1976; Gunnarson et al., 2012), we used climate data from May of the previous year to September of the current year of growth for the analyses. Additionally, we calculated the Expressed Population Signal (EPS), the mean interseries correlation between different trees (Rbar_bt_), and the signal‐to‐noise ratio (SNS).

Our initial exploratory data analysis showed the start of an upward trend in both Scots pine growth and mean annual temperature in the 1980s. Prior to this, temperature declined over approximately four decades and we therefore decided on a 120‐year‐long study period that we divided into three equally long consecutive time periods (1902–1941, 1942–1981, and 1982–2021). Due to the lower age of the downy birch trees, we only calculated the climate–growth relationships for the latter two time periods for a subset of 26 trees that crossdated well.

## 3 RESULTS

Across the eight micro‐sites, the mean age (±SD) ranged from 70 (±51) to 182 (±88) for Scots pine trees and from 84 (±27) to 114 (±33) years for downy birch trees (Table 1). Figures show the diameter distributions for all sampled trees as well as the sample depths, that is, how many samples were included in the analyses in a certain year, which can be found in the supplementary material (Figures S1–S3).

### 3.1 Changes in mean annual BAI and growth trends across different micro‐sites from 1902 to 2021

**FIGURE 3:**
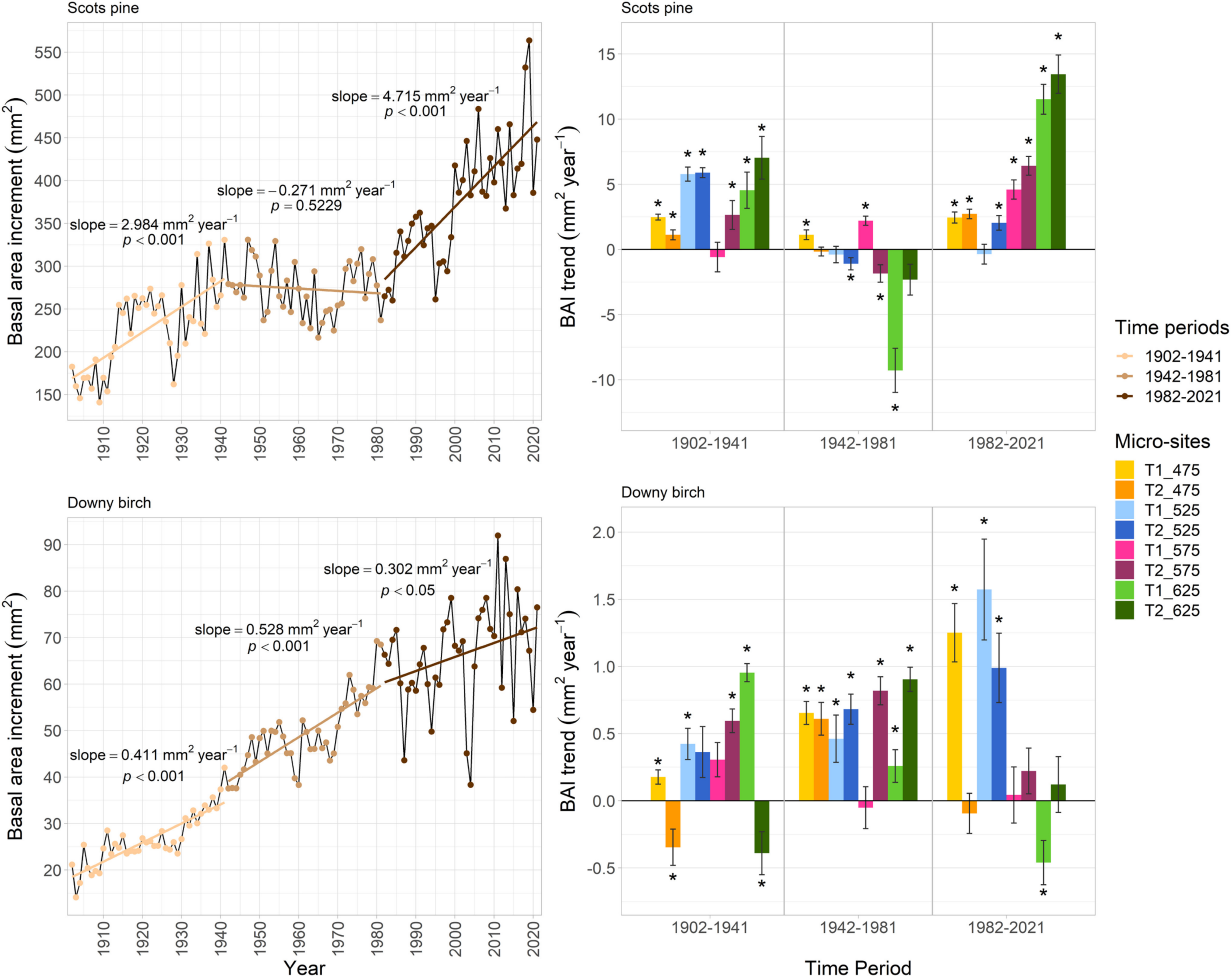
Left: Annual mean basal area increments (BAI) of Scots pine and downy birch across all micro‐sites in Tjeggelvas nature reserves. The slope of the trend lines for the three different time periods shows the mean increase or decrease in BAI compared with the previous year. The steeper the positive slope, the more BAI increased from 1 year to the next. Right: BAI trends for 1902–1941, 1942–1981, and 1982–2021. The heights of the bars refer to the steepness of the linear regression, and the asterisks indicate significant growth trends (*p* < .05). Error bars indicate the standard deviation of values from the regression line.

Overall, the mean Scots pine BAI more than doubled (183 to 448 mm^2^) while the mean downy birch BAI increased more than three‐fold (21 to 77 mm^2^) from 1902 to 2021 (Figure 3). Scots pine BAI increased significantly during 1902–1941 (2.984 mm^2^ year^−1^; *p* < .001), declined by 0.271 mm^2^ year^−1^ during 1942–1981 and then increased significantly again during 1982–2021 (4.715 mm^2^ year^−1^; *p* < .001). Downy birch BAI increased significantly during all three time periods (1902–1941: 0.411 mm^2^ year^−1^, *p* < .001; 1942–1981: 0.528 mm^2^ year^−1^, *p* < .001; 1982–2021: 0.302 mm^2^ year^−1^, *p* < .05) with more variation in the annual BAI during the latest time period compared to the beginning and middle of the 19th century. The positive growth trends for Scots pine during 1902–1941 and 1982–2021 were consistent across the micro‐sites and the negative trend during 1942–1981 was mostly driven by T1_625 at the highest elevation (−9.279 mm^2^ year^−1^) and coincided with lower mean annual temperatures (Table 1). During the most recent time period, the positive growth trends were strongest in the treeline micro‐sites (T1_625: 11.520 mm^2^ year^−1^; T2_625: 13.445 mm^2^ year^−1^) and overall stronger than during 1902–1941 for the majority of sites. Contrary to Scots pine, the growth trends of downy birch during the latest time period are strongly positive at lower elevations. At the coniferous treeline, the annual growth decreased significantly in one micro‐site (T1_625: −0.460 mm^2^ year^−1^) and was only slightly positive in the other (T2_625: 0.121 mm^2^ year^−1^).

### 3.2 The influence of precipitation and temperature on the mean ring width index

The Expressed Population Signal (EPS) reached a value very close to or greater than 0.85 after 1897 for all Scots pine chronologies, however, none of the downy birch chronologies ever reached an EPS value of 0.85 which made it unlikely to find any climate signal. An overview of the characteristics of the chronologies for each micro‐site (EPS, Rbar_bt_, and SNR) can be found in the supplementary material (Table S2).

**FIGURE 4:**
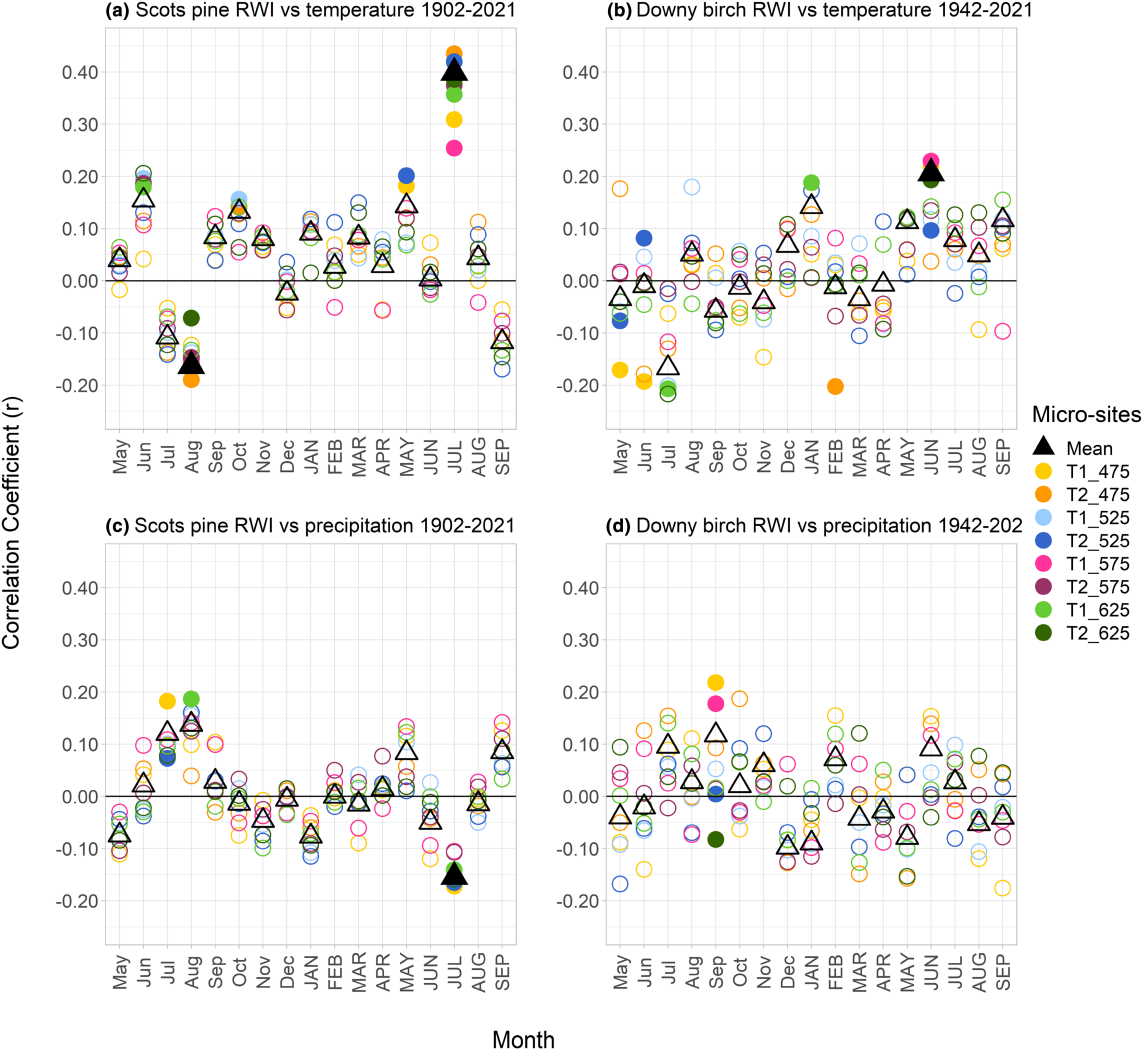
Static correlation coefficient between the mean temperature of the months of the previous year (proper case letters) and the current year (uppercase letters) and the mean ring‐width indices (RWI) of the current year for (a) Scots pine and (b) downy birch growing in the different micro‐site as well as the mean correlations. The correlations between precipitation sums and RWI are shown in (c) for Scots pine and (d) for downy birch. Significant correlations are marked with filled symbols (*p* < .05).

Across the different micro‐sites, the relationships between temperature and RWI were more consistent and significant for Scots pine than for downy birch (Figure 4). There were high correlations between mean July temperature of the current year and RWI at all micro‐sites and a slight increase in strengths of the climate–growth relationships with elevation along Transect 2 (from *r* = .25 at the lowest to *r* = .39 at the highest micro‐site). While the correlation coefficient was also lowest at the lowest micro‐site (*r* = .31), the highest value along Transect 1 was found in T1_525 (*r* = .44) rather than in the treeline micro‐site T1_625 (*r* = .42). There was little to no pattern to the temperature–growth relationships of downy birch trees. Their RWI were positively correlated with mean June temperature across all sites, however, these relationships were much weaker than for Scots pine.

The precipitation–growth relationships were generally weaker than the temperature–growth relationships for both Scots pine and downy birch. Scots pine RWI were negatively correlated with the mean July precipitation sum of the current year in most micro‐sites. As with temperature, there were no clear correlation patterns between mean precipitation sums and downy birch RWI.

### 3.3 Changes in climate–growth relationships over time

**FIGURE 5:**
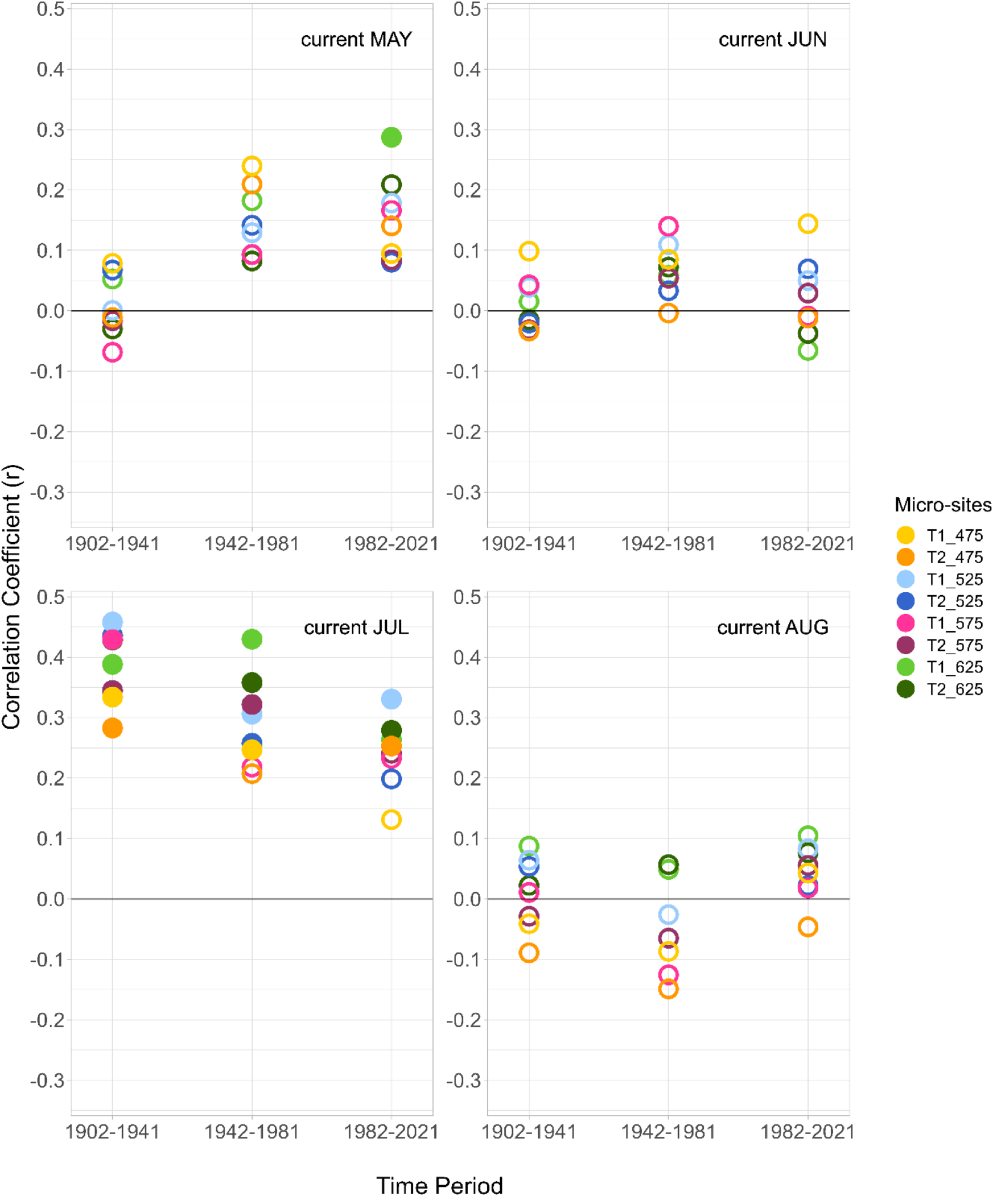
Static correlation coefficients between mean temperatures of May–August of the current year and the mean Scots pine ring‐width indices (RWI) for the three different time periods across the eight micro‐sites. Significant correlations are marked with filled circles (*p* < .05).

We only analyzed the climate–growth relationships of Scots pine over time since we did not find a strong common climatic driver of downy birch RWI across the different micro‐sites. Here, we focus on the correlations between mean temperatures of the growing season of the current year and RWI (Figure 5), figures showing the climate–growth relationships of all months (previous May–current September) can be found in the supplementary material (Figures S4–S7).

The correlations between mean July temperatures of the current year and RWI consistently decreased over time in most micro‐sites, with a notable decline in the number of significant correlations. While correlations were significant in all eight micro‐sites from 1902 to 1941, the number decreased to just three micro‐sites from 1982 to 2021. Conversely, there was an increase in the correlations between mean May temperatures of the current year and RWI, particularly in the high‐elevation micro‐sites. Correlations between the precipitation sums of the current year and RWI were not significant (Figure S4 in the supplementary material).

## 4 DISCUSSION

We assessed the growth of co‐existing Scots pine and downy birch in a natural forest and found increasing growth rates from 1902 to 2021 for both species. However, the correlation between the standardized annual ring width and monthly temperature and precipitation data varied between the two species, with clearer and stronger climate–growth relationships across different micro‐sites for Scots pine.

### 4.1 Changes in mean annual BAI and growth trends across different micro‐sites from 1902 to 2021

We found highest Scots pine BAI (and most positive BAI trend) at higher elevations which is in line with Franke et al. (2017) who also found higher annual growth, expressed as mean ring width index, along the pine treeline than in the forest zone at lower elevations in Finland. They attribute this discrepancy to increased competition at lower elevations that especially affect young trees. Aakala et al. (2018) found positive birch and pine BAI trends in old‐growth forests in Finland as well and their results also show that the growth of pine and spruce was positively influenced by elevation which is in line with our findings. The increase in pine growth we observed over time in TNR is contrary to Linderholm (2002) who found that in the central Swedish mountains, it peaked in the 1950s and has declined since. However, since the main objective of their study was to prolong the local pine chronology rather than study tree growth patterns, there was a bias toward sampling old and big trees which could be the reason for the apparent decline in growth. They also speculate that younger trees might be better at adjusting to climate change. This age effect, combined with a less dense forest and hence a decreased importance of competition at higher elevations (Callaway et al., 2002), might allow the Scots pine trees to take full advantage of increasing temperatures. This would explain the strong increase in annual growth we found at the highest micro‐sites. However, we also recognize that our study might be biased by the comparatively young trees growing at higher elevations.

While the pines along the treeline grew especially well during the latest time period, the growth trend was strongly negative in T1_625 from 1942 to 1981 (−9.279 mm^2^ year^−1^). This anomaly might be linked to the regeneration pulses of Scots pine (Zackrisson et al., 1995), the negative temperature trend during that time period (Figure 1), or a combination of both. While the number of trees in T1_625 increased strongly until the 1970s, there was no abrupt increase in the number of trees around that time in the other treeline micro‐site T2_625 (Figure S2 in the supplementary material). The strongly positive trends in BAI visible in both treeline micro‐sites during the latest time period can therefore not be explained by regeneration pulses or tree age alone; Scots pine growth rates were also higher in T2_625 than in T1_625 even though the mean tree age there is 24 years lower (Table 1).

Decreasing competition between Scots pine trees with elevation, evidenced by the comparatively low number of stems per hectare at the coniferous treeline (Table 1), in the subalpine forest in TNR could possibly explain the high growth rates in these two treeline micro‐sites and the drastic increase in the number of Scots pine trees in T1_625 after the 1960s. The downy birch density in this particular micro‐site was much higher than in any other micro‐site (T1_625: 481 stems/ha; T2_625: 56 stems/ha; see Table 1) and the facilitative effect of birch on pine along the coniferous treeline has already been shown by Mikola et al. (2018). Soils are typically better under deciduous trees due to faster nutrient cycling (Kanerva & Smolander, 2007) and the resulting increased availability of nitrogen could play a role in why Scots pine trees establish in certain spots along the treeline but not in others.

Interestingly, the downy birches did not grow so well where the Scots pine trees grew the best (Figure 3). This result suggests that increasing competition between the two species due to environmental stress might have already started to outweigh the facilitative effect of downy birch on Scots pine along the coniferous treeline.

### 4.2 The influence of precipitation and temperature on the mean ring‐width index

Low EPS and Rbar_bt_ values (Table S2 in the supplementary material) showed that the climate signal retained in the ring widths of downy birch was not very strong. However, Scots pine growth was strongly influenced by the climate with temperature having a more pronounced effect than precipitation.

There was a high correlation between mean July temperature of the current year and RWI which showed that Scots pine trees benefitted most from the July temperatures during their growing season, that is, the greater the mean temperature, the greater the RWI. This is in line with previous studies conducted in Fennoscandia (e.g., Franke et al., 2017; Tuovinen, 2005; Young et al., 2011) and confirms that growing season temperature is the main growth‐limiting factor at our sites.

While Gunnarson et al. (2012) found stronger climate–growth relationships in TNR for Scots pine, this can be explained by our different sampling methods of coring every single living tree within our micro‐sites. For studies focusing solely on dendroclimatology, it is important to select trees that depend as little on nonclimatic factors as possible, which are usually the straight, dominant trees in a stand (Fritts, 1976; Schweingruber, 1988). However, since we used a more ecological sampling approach which is more representative of the whole forest (Nehrbass‐Ahles et al., 2014), this also means that competition and specific site characteristics have a greater influence on our results than if we had just cored the dominant trees, therefore lowering correlation values with climate variables.

The strength of the relationship between Scots pine RWI and temperature also slightly increases with increasing elevation. Düthorn et al. (2013) found that the influence of summer temperature on Scots pine RWI varies depending on micro‐site conditions. We also found differences in the strengths of the climate–growth correlations between different micro‐sites, which highlights the importance of topography and micro‐climate on RWI on a small scale.

Similar to Young et al. (2011), we also found a negative correlation between mean July precipitation and annual Scots pine RWI, which could be explained by an increased cloud cover and therefore less sunshine and photosynthesis (Fritts, 1976).

The higher signal‐to‐noise ratios (SNR) and lower between‐tree correlations (Rbar_bt_) (Table S2 in the supplementary material) for downy birch compared to Scots pine trees indicated that the growth patterns of individual birch trees were not very similar even if they grew in close proximity to each other (Speer, 2010). This might mean that the downy birches are more dependent on micro‐site conditions (e.g., soil moisture, nutrient availability, browsing) than the Scots pine trees, which could also explain the inconsistent climate–growth relationships across the different micro‐sites. Additionally, autumnal moth (*Epirrita autumnata*) outbreaks regularly occur in northern Sweden (Young et al., 2014) which also affects the growth patterns.

Only the correlation between the downy birch RWI of our 26 samples and the mean June temperature was significant in our study, whereas other studies found both June and July temperatures to be important for downy birch growth in northern Sweden (Eckstein et al., 1991; Karlsson et al., 2004; Young et al., 2011) and northern Iceland (Levanič & Eggertsson, 2008). In northern Norway, May and June temperatures are most crucial (Harr et al., 2021).

Downy birch is characterized by indeterminate growth (Weih, 2000) which likely contributes to the greater importance of early growing season temperatures. In contrast, Scots pine exhibits strong determinate growth and is therefore also influenced by the growing conditions of the previous years (Tuovinen, 2005). Early growing season conditions are therefore less important for Scots pine than downy birch growth, which is evidenced by the non‐significant of mean June temperatures for Scots pine growth.

### 4.3 Changes in Scots pine climate–growth relationships over time

Düthorn et al. (2016) found a decreasing importance of summer (July and August) temperature and slightly increasing importance of summer precipitation for the latewood density of Scots pine in Finland from 1901 to 2011. While the influence of previous years' conditions is greater on tree‐ring width than the latewood density, possibly making the relationships between climate and tree‐ring widths less clear (Büntgen et al., 2010), their results are still partly in line with what we found: In Tjeggelvas, mean July temperature has become less important for pine RWI over time while the influence of mean August temperature has been consistently low. This could, particularly at higher elevations, be due to increasing competition limiting the positive influence of global warming on growth. Alternatively, the combination of the decreasing importance of mean July temperatures and the increase in BAI during most recent times could also mean that growing conditions are becoming more favorable, particularly at higher elevations.

One of the most intriguing findings is the strong increase in the importance of mean May temperature on Scots pine RWI in micro‐site T1_625. This could again, like the high annual growth in this micro‐site, be linked to the exact growing locations of the pines. We speculate that the Scots pine trees might have either established in depressions where they are less exposed to wind and there is more moisture available during the summer due to later snowmelt (Johnson & Yeakley, 2019) or that they benefit from earlier snowmelt due to higher temperatures in May.

While small changes in the timing of the snowmelt in the Arctic tundra would probably not have a big impact on microbial activity and nutrient cycling (Darrouzet‐Nardi et al., 2019), this might be different in subarctic forests with higher temperatures in May leading to earlier snowmelt and higher soil temperatures. As an indirect effect of an increase in temperature, the increased nutrient availability due to higher rates of litter decomposition (Rustad et al., 2001) could have also contributed to the higher Scots pine growth rates along the coniferous treeline.

### 4.4 The larger perspective—Climate and tree growth in protected and managed forests in northern Sweden

Understanding both the growth and the climate–growth relationships in natural forests in northern Sweden is crucial as many of the forests close to the Fennoscandian mountain range are protected. Additionally, substantial areas of formerly commercial forests have recently been protected (Felton et al., 2020) or are currently in the process of being protected as new forest reserves. Therefore, the response of these large areas of natural forests to climate change is important to evaluate.

In contrast, however, there is an increasing loss of semi‐natural forests outside reserves due to the intensification of forestry in recent times (Svensson et al., 2019). To further complicate matters, the management of commercial forests in northern Sweden is at a stage of transition due to external pressure from conservationists and indigenous Sámi reindeer herders. The demands are toward less intensive methods such as continuous cover forestry, retention of old trees, and the use of natural disturbances as models for near‐nature management methods (cf. Kuuluvainen et al., 2021).

We found a strong increase in Scots pine growth in recent years, which contrasts with the Scots pine growth decline in northern Sweden found by the NFI (Fridman et al., 2022) and underscores the complexity of the impacts of global warming on forest ecosystems. In light of this discrepancy, it is crucial to understand how climate change affects natural forest ecosystems per se but also to use this knowledge for improved management of the commercial forests in the region.

## 5 CONCLUSIONS

Our study focused on the Scots pine/downy birch forest transition in a northern forest reserve. The analyses showed an increased tree growth for both species and particularly so during the most recent decades. The continued increase in the growth (and thus aboveground carbon storage) contrasts recent developments in managed forests. Since northern Sweden contains many of the largest protected forests in the country, their response to climate warming has significance for carbon budgets, emphasizing the need to understand their development. We found that climatic conditions were more crucial for Scots pine than downy birch and temperature more so than precipitation across all elevations. Summer temperatures were important for the growth of both species with June temperatures having the greatest effect on downy birch growth and July temperatures being most important for the Scots pine trees. However, the importance of the mean July temperature decreased over time. We suggest that more research is needed to understand the effect of micro‐climate and specific site conditions on birch growth as well as inter‐species relationships in mixed natural forests. Furthermore, we believe that natural forests along the Fennoscandian mountain range are important natural experiments, in contrast to the managed forest landscapes in Sweden and a key to understanding and improving the management of the latter. Overall, due to strongly positive BAI trends combined with a decrease in temperature sensitivity, the influence of increasing temperatures is still positive and outweighs the negative impacts of climate change on Scots pine growth in natural forests in northern Sweden, particularly at higher elevations.

## FUNDING INFORMATION

This study was funded by Anna och Nils Håkanssons Stiftelse, Östersund.

## CONFLICT OF INTEREST STATEMENT

The authors declare that they have no conflict of interest.

## Supporting information


Data S1.

## Data Availability

The data is available from the corresponding author upon request.
