# Supplementary material for: Elevation‐dependent tree growth response to climate in a natural Scots pine/downy birch forest in northern Sweden
Source: Plant Environ Interact. 2024 Apr 1;5(2):e10140. doi: 10.1002/pei3.10140 (PMC10984726; doi:10.1002/pei3.10140)
Supplement: Supplementary file 1 — Data S1. [file PEI3-5-e10140-s001.docx]

**Elevation-dependent tree growth response to climate in a natural Scots pine/downy birch forest in northern Sweden**

**Supplementary material**

Magdalena Fassl^1*^, Tuomas Aakala^2^, Lars Östlund^1^

1) Department of Forest Ecology and Management, Swedish University of Agricultural Sciences, Umeå, Sweden

2) School of Forest Sciences, University of Eastern Finland, Joensuu, Finland

*corresponding author: **magdalena.fassl@slu.se**

Many of the largest Scots pine trees had rotten centers and could therefore not be included in the BAI analyses (Figure S1). The majority of sampled downy birch trees had diameters between 8.0 and 12.5 cm at breast height.

**
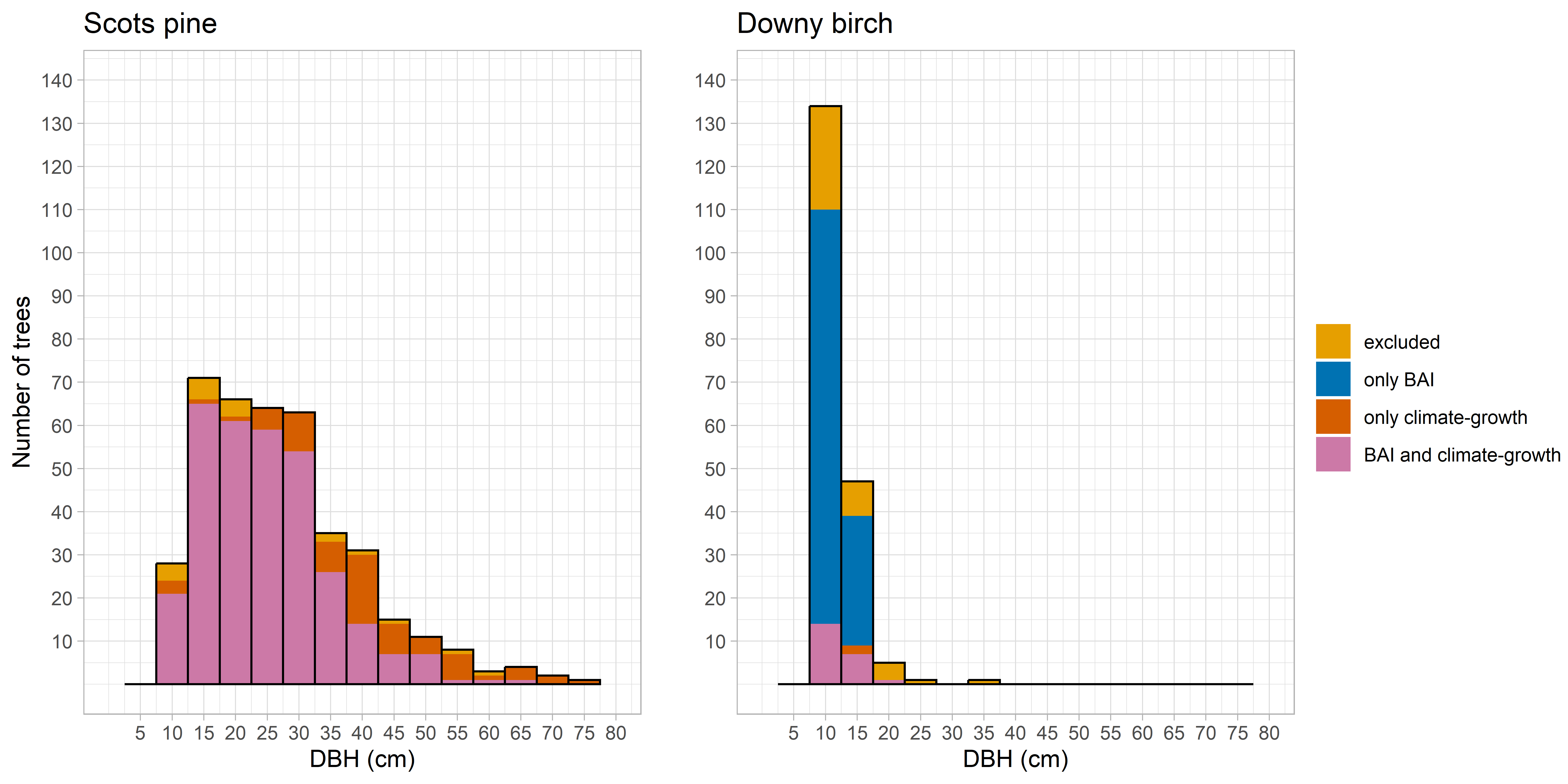
**

Figure S1. DBH distributions of all sampled Scots pine and downy birch trees in TNR. The bin size is 5 cm.

The sample size generally increased until the 1990s. There was a particularly drastic increase in the number of Scots pine trees in micro-site T1_625 during the 1960s and 1970s (Figure S2).


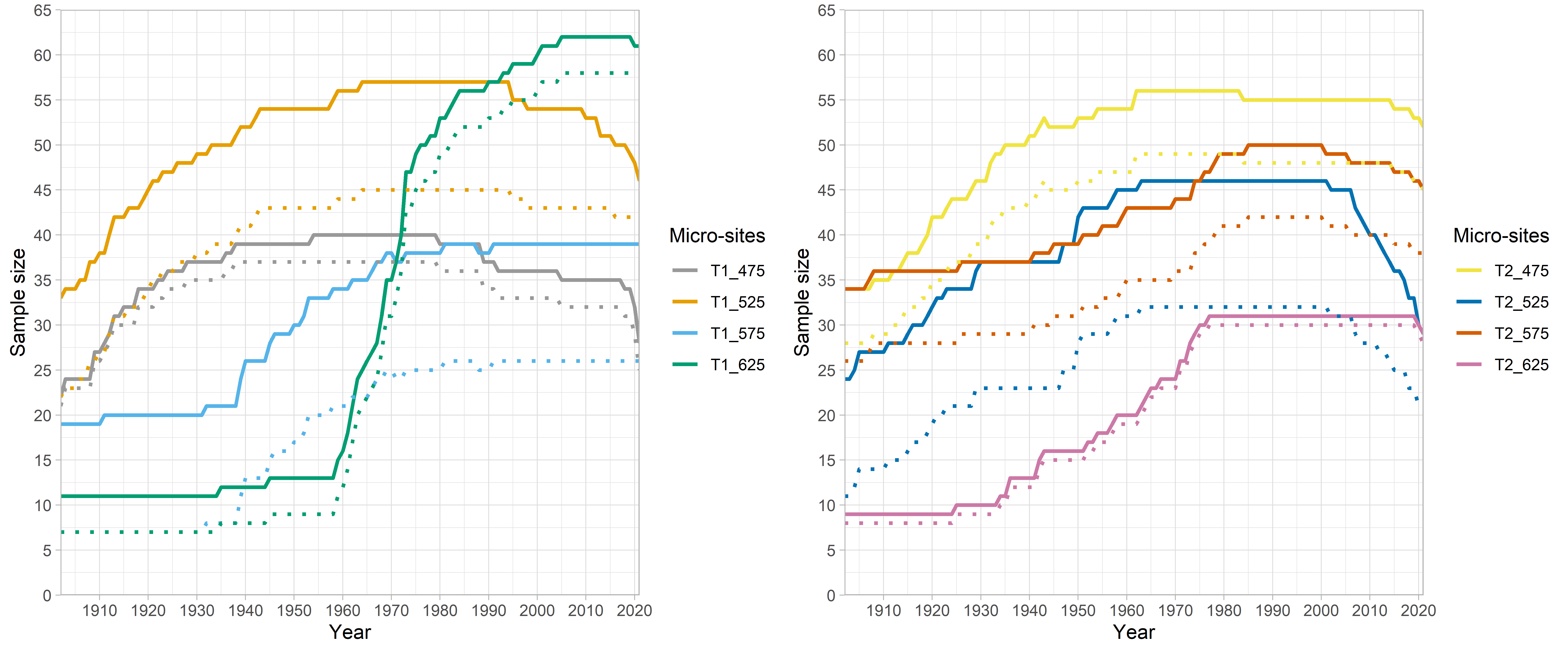


Figure S2. Sample size of Scots pine trees over time in the different micro-sites. Solid lines represent the number of trees included in the climate-growth analyses, dotted lines the number of trees included in the BAI analyses.

Although all trees were still alive at the time of sampling, there was less data available for the most recent years in certain micro-sites as some of the increment cores broke and the outermost parts were lost during the fieldwork. This was especially an issue with downy birch cores in micro-site T2_625 (Figure S3).


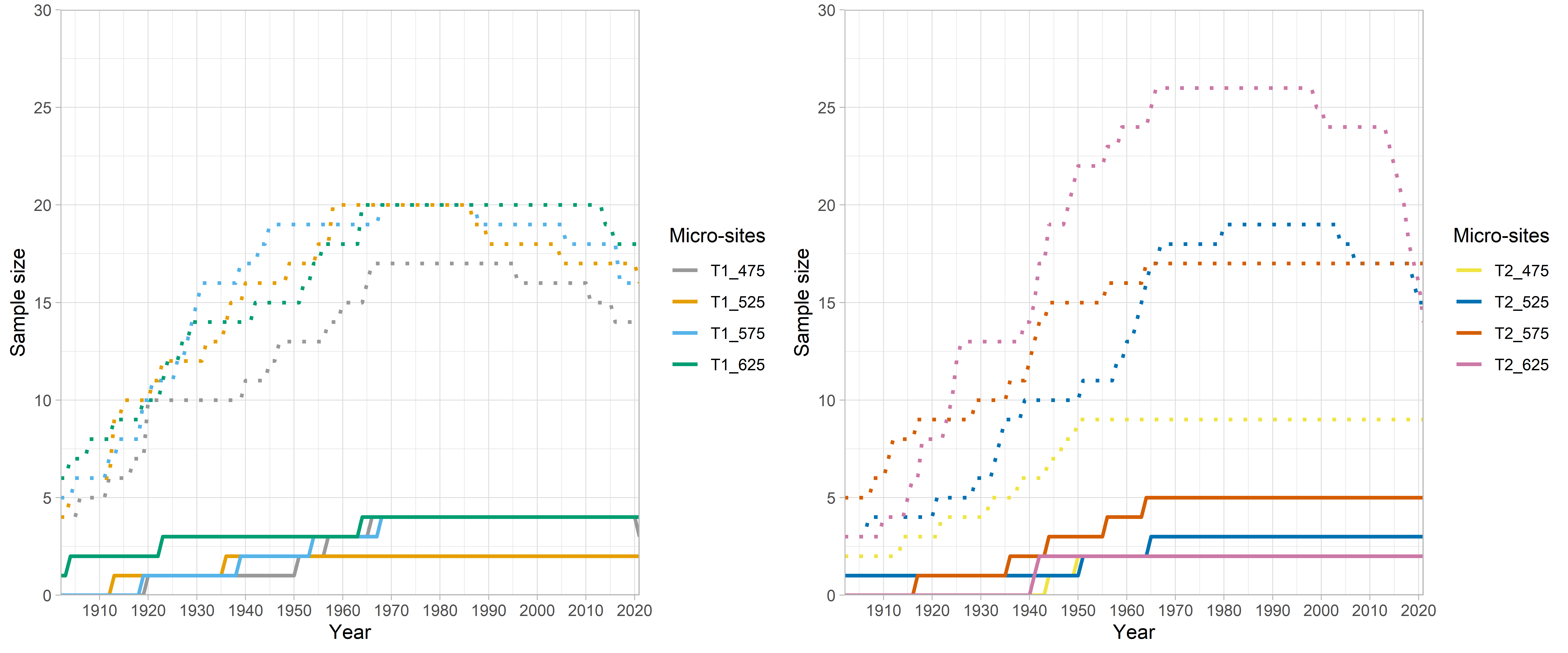


Figure S3. Sample size of downy birch trees over time in the different micro-sites. Solid lines represent the number of trees included in the climate-growth analyses, dotted lines the number of trees included in the BAI analyses.


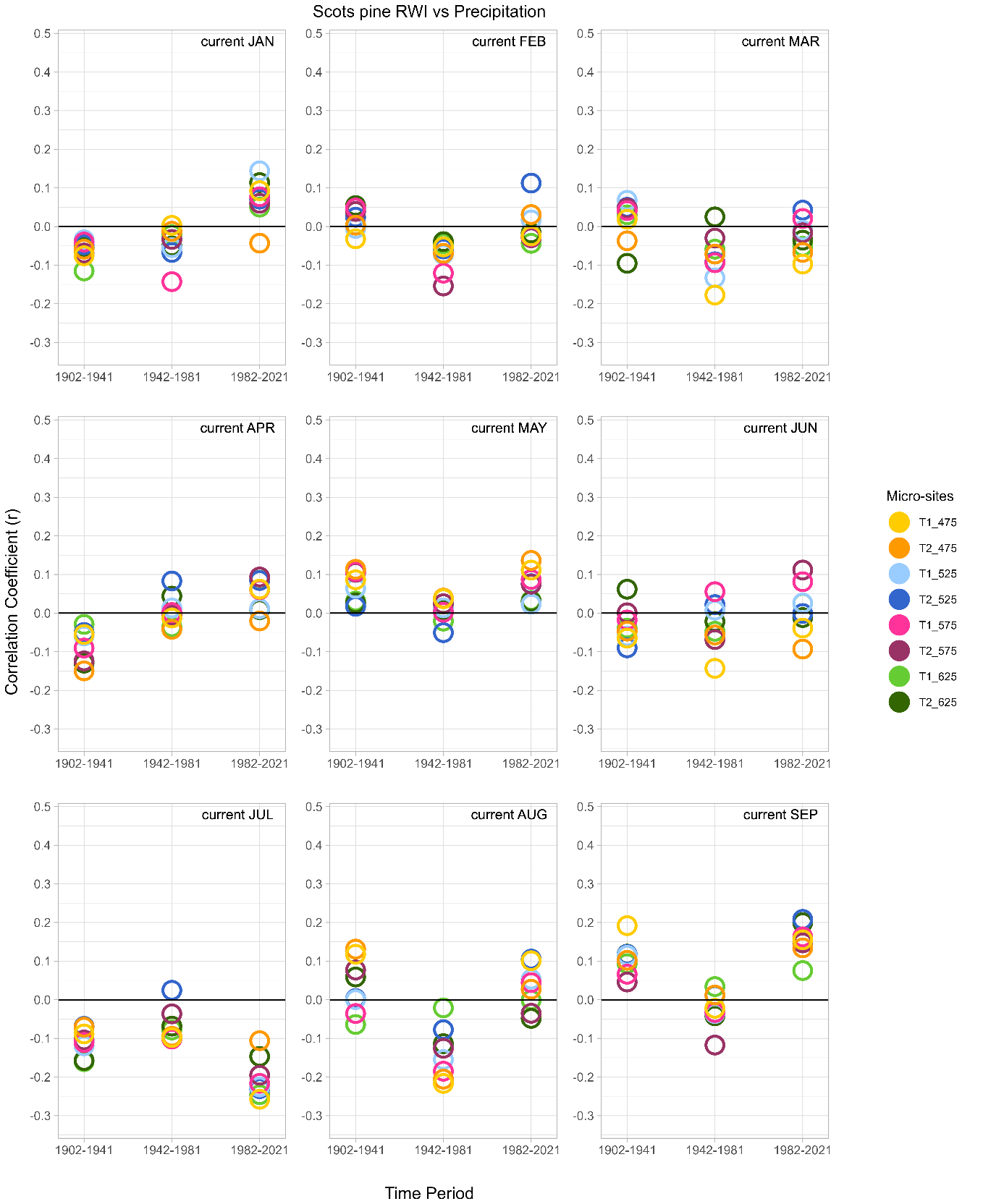


Figure S4. Static correlation coefficients between the precipitation sums of the current year and the mean Scots pine ring width indices (RWI) for the three different time periods across the eight micro-sites. Significant correlations are marked with filled circles (*p* < 0.05).


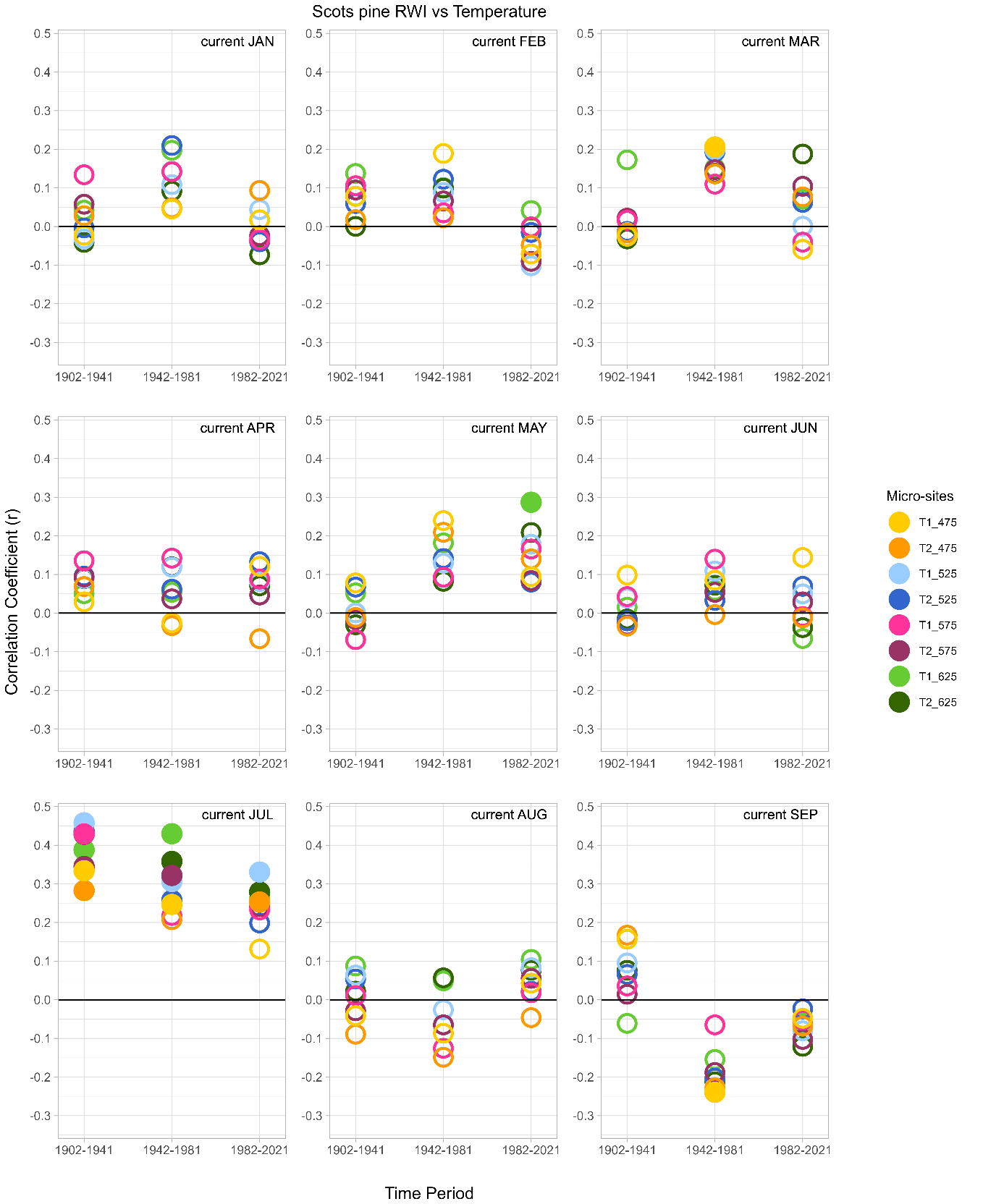
Figure S5. Static correlation coefficients between the mean monthly temperatures of the current year and the mean Scots pine ring width indices (RWI) for the three different time periods across the eight micro-sites. Significant correlations are marked with filled circles (*p* < 0.05).


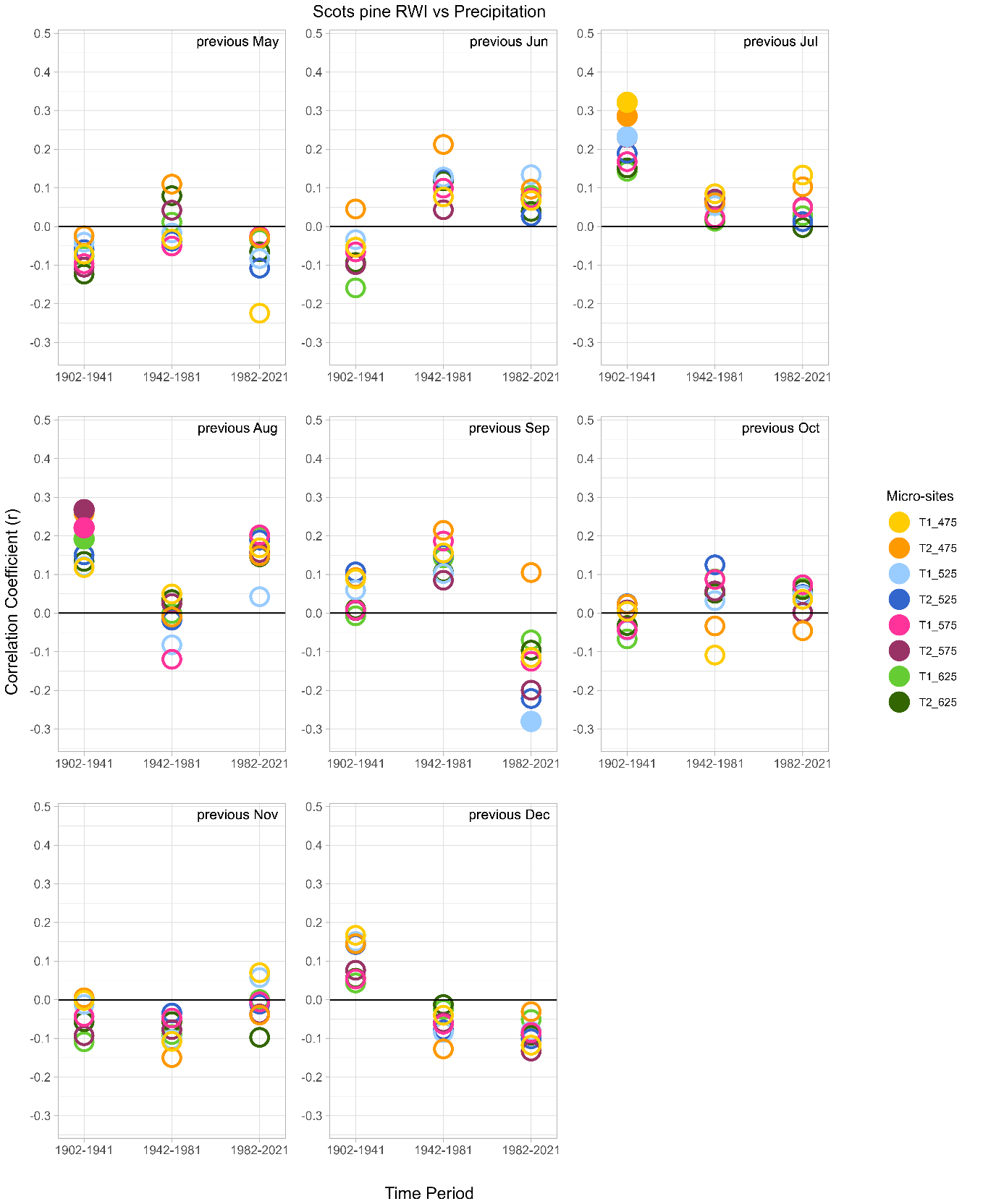
Figure S6. Static correlation coefficients between the precipitation sums of the current year and the mean Scots pine ring width indices (RWI) for the three different time periods across the eight micro-sites. Significant correlations are marked with filled circles (*p* < 0.05).


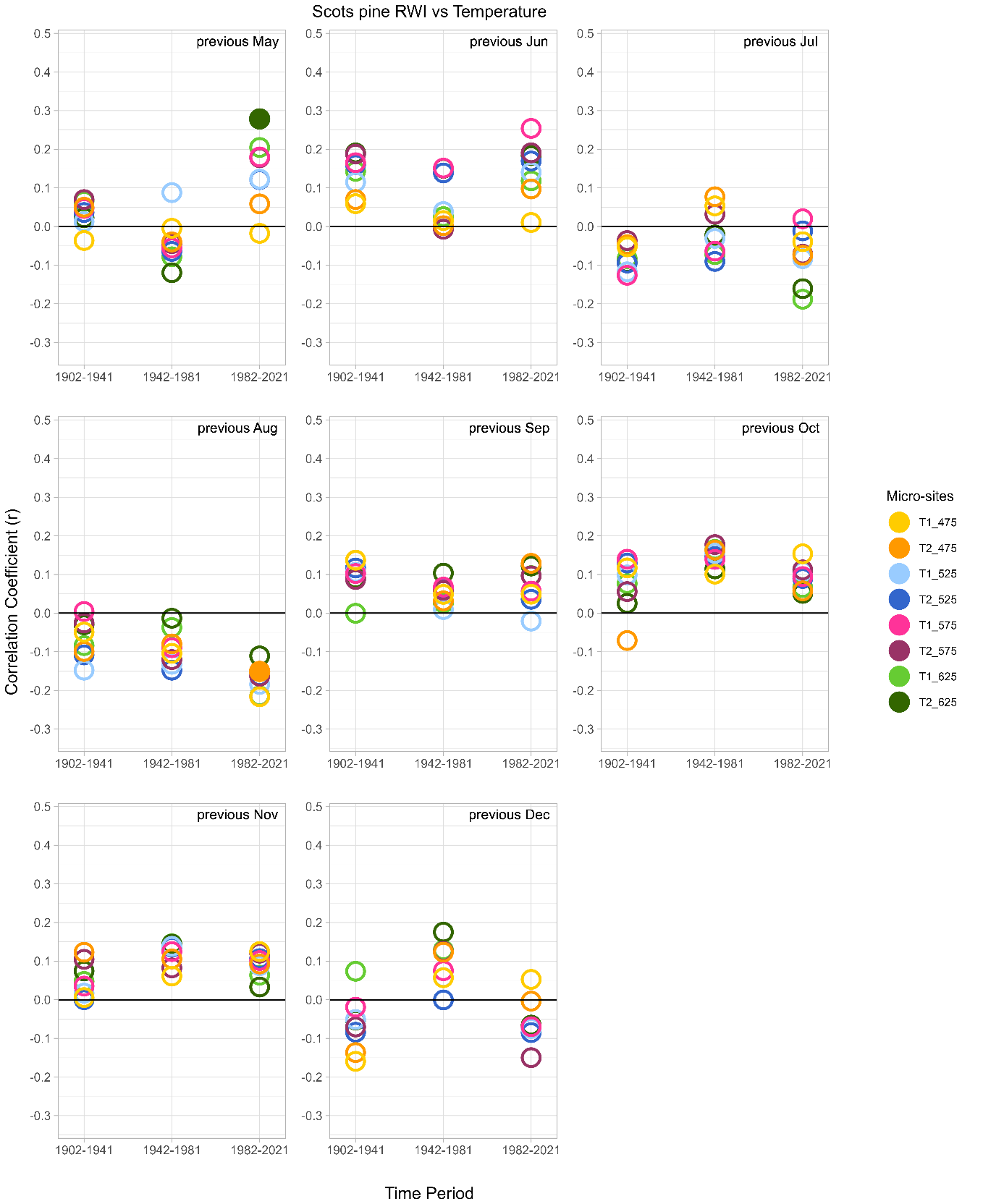


Figure S7. Static correlation coefficients between the mean monthly temperatures of the previous year and the mean Scots pine ring width indices (RWI) for the three different time periods across the eight micro-sites. Significant correlations are marked with filled circles (*p* < 0.05).

Table S1. The number of stems refers to the number of living trees with a diameter at breast height of at least 10 cm for Scots pine and 8 cm for downy birch within each micro-site.

| Micro-site | Micro-site radius  [Scots pine / downy birch] | Number of stems in micro-site  [Scots pine / downy birch] | Dominant ground vegetation |
| --- | --- | --- | --- |
| T1_475 | 25 / 25 | 45 / 25 | Lichen, dwarf shrubs |
| T2_475 | 30 / 30 | 61 / 10 | Dwarf shrubs |
| T1_525 | 30 / 30 | 58 / 28 | Dwarf shrubs |
| T2_525 | 25 / 45 | 50 / 22 | Dwarf shrubs |
| T1_575 | 25 / 20 | 43 / 27 | Lichen, dwarf shrubs |
| T2_575 | 40 / 30 | 44 / 21 | Lichen |
| T1_625 | 50 / 15 | 63 / 24 | Lingonberry |
| T2_625 | 50 / 50 | 30 / 34 | Lichen, dwarf shrubs |

Table S2. Characteristics of the chronologies for each micro-site. Rbar_bt_ is the between-tree correlation, EPS the expressed population signal and SNR the mean signal-to-noise ratio. The values were calculated for the full periods covered by the chronologies in the different micro-sites.

|  | Micro-site | Mean Rbar_bt_ (between-tree correlation) | Mean EPS | EPS > .85 after … | Mean SNR |
| --- | --- | --- | --- | --- | --- |
| Scots pine | T1_475 | 0.226 | 0.921 | 1897 | 11.687 |
|  | T2_475 | 0.117 | 0.883 | 1897 | 7.532 |
|  | T1_525 | 0.280 | 0.957 | 1847 | 22.153 |
|  | T2_525 | 0.188 | 0.914 | 1872 | 10.670 |
|  | T1_575 | 0.250 | 0.932 | 1847 | 13.659 |
|  | T2_575 | 0.234 | 0.939 | 1847 | 15.301 |
|  | T1_625 | 0.280 | 0.957 | 1922 | 22.156 |
|  | T2_625 | 0.322 | 0.936 | 1897 | 14.744 |
|  |  |  |  |  |  |
| Downy birch | T1_475 | 0.259 | 0.583 | - | 1.400 |
|  | T2_475 | 0.402 | 0.573 | - | 1.343 |
|  | T1_525 | 0.258 | 0.410 | - | 0.695 |
|  | T2_525 | 0.344 | 0.612 | - | 1.576 |
|  | T1_575 | 0.187 | 0.479 | - | 0.919 |
|  | T2_575 | 0.426 | 0.789 | - | 3.710 |
|  | T1_625 | 0.246 | 0.566 | - | 1.304 |
|  | T2_625 | 0.662 | 0.796 | - | 3.911 |
